# Coarse-grained molecular dynamics investigation on the interaction between κ- and β-casein aggregates and curcumin

**DOI:** 10.1371/journal.pone.0328010

**Published:** 2025-07-10

**Authors:** Jian Gao, Rong Dong, Shuailing Yang, Zhengtao Zhao, Ye-Wang Zhang

**Affiliations:** 1 School of Grain Science and Technology, Jiangsu University of Science and Technology, Zhenjiang, People’s Republic of China; 2 Jiangsu Provincial Engineering Research Center of Grain Bioprocessing, Zhenjiang, People’s Republic of China; 3 School of Pharmacy, Jiangsu University, Zhenjiang, People’s Republic of China; Yantai Institute of Technology, CHINA

Caseins, the predominant proteins in milk, play a crucial role in the formation of micellar complexes due to their amphiphilic nature, which facilitates the formation of micellar complexes, with κ-casein stabilizing micelle formation. Understanding the self-assembly of casein molecules is essential for exploring their potential applications, such as nanoencapsulation of bioactive compounds. This study delves into the structural properties and self-assembly mechanisms of κ-casein and β-casein using computational models and simulations. Through coarse-grained (CG) modeling, the study investigates the stable structure of an aggregate composed of -casein and β-casein after 1 μs of CG simulation, highlighting a loose arrangement with water molecules in the cavity. Simulations of the encapsulation process for the bioactive compound show that the curcumin tends to adhere to the surface of the casein aggregate and cannot spontaneously be encapsulated by casein. Additionally, Steered Molecular Dynamics (SMD) simulations shed light on the interaction between the aggregate and curcumin, illustrating the challenges faced by insoluble compounds in entering casein micelles due to the tightly packed arrangement of κ-casein. Based on the simulation results, the curcumin can’t penetrate the casein aggregate spontaneously. External forces, such as homogenization and ultrasonication, are required to overcome the energy barrier and facilitate the complexation of curcumin with caseins. These findings provide theoretical guidance for using caseins as encapsulation agents for hydrophobic functional compounds.

## 1. Introduction

Caseins are the predominant proteins in milk, accounting for approximately 80% of its total protein content [1,2]. Bovine milk contains four primary casein types: α_S1_-, α_S2_-, β-, and κ-casein, present in a molar ratio of 4:1:3.5:1.5. In human milk, micelles are composed of α_S1_-, β-, and κ-casein in a ratio of 12:68:20, with α_S2_-casein being undetectable [3]. The amphiphilic nature of caseins, characterized by hydrophobic clusters and negatively charged regions along their peptide chains, facilitates the formation of large colloidal aggregates that lead to the assembly of micellar complexes. These protein assemblies exhibit a strong affinity for bivalent and trivalent cations, particularly through interactions at phosphoserine residues, such as those found in colloidal calcium phosphate nanoclusters, which contribute to the formation of casein micelles.

From a molecular perspective, caseins are rich in proline, a feature that inhibits the formation of globular structures, resulting in predominantly random-coiled conformations lacking folded structure [1]. This lack of tertiary structure confers high intramolecular flexibility, enabling caseins to self-assemble into core-shell structures in aqueous environments. The structural properties of reassembled micelles can be influenced by various factors, including concentration [4], temperature [5], ionic strength [1], and pH [6]. The mechanism of casein micelle formation has been described previously [7,8]. However, the precise arrangement of packing within the casein micelles remains an area of active debate. Several models have been proposed to describe the structure of casein micelles, including the submicelle model [9,10], the nanocluster model [11], and the dual-binding model [12]. Among all these models, the nanocluster model is the most widely accepted. According to this model, α_S_- and β-caseins combine with calcium phosphate to form the internal structure, while κ-casein, which lacks a phosphate group, is located on the surface, providing steric and electrostatic repulsions that stabilize the micelle [13]. Observations indicate that the interior of casein micelles is not homogeneous but has a porous structure [14], which enables it to encapsulate a variety of bioactive compounds [9–11]. Unlike their globular counterparts, caseins can interact with multiple targets, and they also exhibit chaperone-like activity, solubilizing hydrophobically aggregated proteins under stress conditions, such as elevated temperatures or reducing environments [15,16]. Native or reassembled casein micelles have been successfully used for nanoencapsulation of hydrophobic bioactive compounds, including β-carotene [17], vitamin D2 [18], α-tocopherol [19], polyunsaturated fatty acids [20],caffeic acid [21], triclosan [22], curcumin [16,23,24], probiotics [25], and quercetin [26,27]. However, it remains controversial whether the interaction between hydrophobic compounds and caseins occurs spontaneously, as well as the preferred binding locations of these compounds on casein micelles.

Hydrophobic interactions are thought to be the primary force driving the encapsulation of bioactive compounds within casein micelles. However, the fundamental mechanisms underlying these interactions require further investigation. Key questions include the extent to which compounds are trapped within the micelles, their preferred locations, and the ease with which they can be released. Additionally, there is interest in understanding whether encapsulation differs when using caseins with varying degrees of hydrophilicity and distribution positions, such as the shell parts of κ-caseins versus the internal core of β-caseins. To advance our understanding of casein self-assembly and address these questions, this research employs computer simulation methods to investigate the structure and micelle formation processes of κ-casein and β-casein. Curcumin is used as a model ingredient to explore changes in the forces between caseins and the bioactive compound as it passes through the micelle. The findings from this study aim to elucidate the formation mechanisms of casein micelles and provide theoretical guidance for the use of β- and κ-caseins as encapsulation materials for functional ingredients.

## 2. Materials and methods

### 2.1 Predicted structure of casein

**Fig 1:**
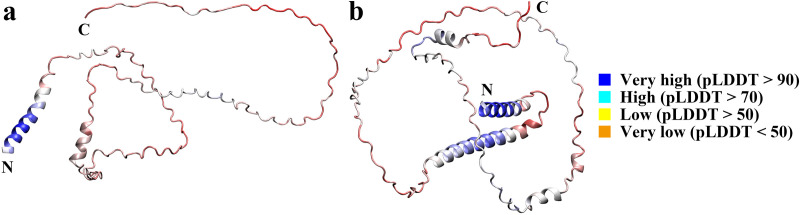
The structure of κ-casein (a) and β-casein (b) from Bos Taurus predicted by AlphaFold (UniProt: P02668 and P02666). The first 21 residues segment of κ-casein and the first 15 residues segment of β-casein are signal peptides. AlphaFold produces a per-residue model confidence score (pLDDT) between 0 and 100. Some regions below 50 pLDDT may be unstructured in isolation.

Casein has no regular tertiary structure. Fig 1 shows the predicted structures of κ-casein (UniProt: P02668) and β-casein (UniProt: P02666) molecules as found in the AlphaFold Protein Structure Database (https://alphafold.ebi.ac.uk/) [28]. The signal peptides of the κ-casein (residues 1-21) and β-casein (residues 1-15) are both helices, as is the residues 16-64 section of β-casein that is linked to the signal peptide. κ-casein and β-casein also have very short fragments predicted to have a helical structure, but the confidence score by the predicted local distance difference test (pLDDT) is low and can be considered irregular coil.

### 2.2 Coarse-gained model

The all-atom model can provide a more accurate description of protein molecules; In particular, it is able to simulate the formation of hydrogen bonds and secondary structures. However, the computational load is huge for an all-atom model to simulate a big complex system. The formation process of an aggregate composed of several casein molecules requires a larger time scale. The coarse-grained (CG) model is more advantageous for simulating mesoscale structures at the cost of losing the information at the atomic level. Among the CG models, the MARTINI model particularly considers the amphiphilic interactions during the formation of compound structures, and has been widely applied in the last two decades to successfully describe many biological processes [29]. In the MARTINI model [30–32], four water molecules are mapped to one water particle, and approximately four or more heavy atoms are usually mapped to one particle. Protein molecules are composed of different types of particles. Compared with the all-atom model, the CG model can simulate larger systems, with faster conformational space sampling speed but reduced accuracy. The MARTINI model was built for κ-casein and β-casein in this work.

**Fig 2:**
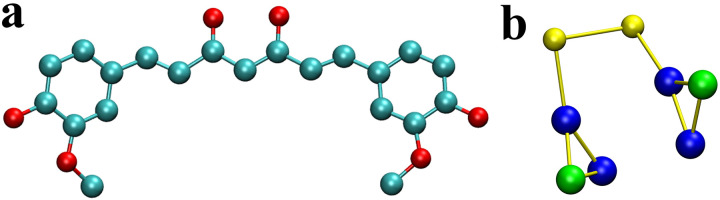
The all-atom (a) and the MARTINI (b) model of curcumin molecule. The carbon atoms are shown in cyan and the oxygen atoms in red in the all-atom model. The “NDAT” beads (yellow) are backbone of curcumin, and the “SC4Y” (blue) and “SP1Y” (green) beads are side chain in the MARTINI model.

The structure of curcumin molecules is shown in Fig 2a, consisting of two symmetrical structures similar to two connected tyrosines (TYRs). Therefore, the MARTINI model we built for curcumin (see Fig 2b) is composed of two TYRs. Considering the non-polarity and turn structure of the curcumin molecule, the backbone particle type is designed as “NDAT”, and the side chain particle types are “SC4Y” and “SP1Y”, just like tyrosine. After removing the signal peptides, the MARTINI models were built for κ-casein and β-casein. Due to the inability of CG models to accurately describe each real atom, they cannot simulate the formation of hydrogen bonds and secondary structures. To reflect the secondary structure of protein molecules, the MARTINI model defines different types of virtual atoms and maintains stable secondary structures through harmonic constraints. The κ-casein and β-casein are charged with -2 and -8, respectively.

### 2.3 Self-assembly simulation of the aggregate

Starting from the structures predicted by AlphaFold, four CG κ-casein and four CG β-casein molecules were assembled in a CG water box using CHARMM [33,34] and forty CG K^+^ ions were added to neutralize the system. The final systems consist of ~87800 water particles besides the casein molecules and ions, with a box size of ~220 × 220 × 220 Å^3^. The NAMD software package [35] was used for simulations because of higher parallel computational efficiency. The equilibrium simulation of 4 ps with a harmonic potential constraint (*k* = 2 kcal/mol/Å^2^) on the backbone particles of casein molecules was performed to eliminate the subtle differences between CHARMM and NAMD. Then, the production simulation of 200 ns at 298 K was carried out. The collapsed final structures of κ-casein and β-casein in water were obtained.

To promote aggregation, these collapsed structures were reassembled into an aggregate composed of nine κ-casein and nine β-casein molecules in a water box to be the initial structure of the self-assembly system. Two self-assembly systems were built with the composition shown in Table 1. An additional nine curcumin molecules were added to the casein-curcumin system to observe whether curcumin molecules could spontaneously embed inside casein aggregates. The equivalent equilibrium simulation setup described above was also used for the self-assembly system. The 1 μs NPT simulation (constant pressure and temperature) with a timestep of 20 fs was carried out to form a stable aggregate. During the simulation, the temperature was kept at 298 K, and the pressure was 1 atm, and a harmonic potential restraint (*k* = 2 kcal/mol/Å^2^) was applied on the backbone particles of the casein to keep the center of the aggregate near the system origin. The Visual Molecular Dynamics (VMD) [36] was used for the preparation of all molecular images presented in this work.

**Table 1:** Composition of two self-assembly simulation systems.

	curcumin	κ- and β-casein	K^+^	H_2_O	size (Å^3^)
Casein	0	9 + 9	90	~17000	~120 × 140 × 140
Casein-curcumin	9	9 + 9	90	~45000	~180 × 180 × 180

### 2.4 Steered Molecular Dynamics (SMD) between casein aggregate and curcumin

The aggregate with κ-casein at the top and β-casein at the bottom was placed at the origin of a water box, and a curcumin molecule was placed at z = 90 Å and x = −90 Å in two SMD simulations, respectively. An external force with a harmonic spring constant of *k* = 2 kcal/mol/Å^2^ was applied to the curcumin to pull it passing through the aggregate along Z- and X-directions, respectively. The constant velocity of 0.005 Å/ps SMD simulations was carried out. In order to avoid moving and distortions of the aggregate as a consequence of interaction with curcumin, the center of the aggregate was restrained with a high force constant of 100 kcal/mol/Å^2^ to constrain the aggregate at the origin of the water box during SMD simulations. In addition, we used a cylindrical harmonic restraint, which avoids curcumin leaving a cylindrical area with a radius of 5 Å and the Z-/X-axis as the central axis. The force constant was the same as the restraint on the aggregate. This restraint allowed curcumin to pass through the gap between casein molecules at the center of the aggregate. Finally, the curcumin molecule was pulled 180 Å distance and passed through the aggregate along Z- and X-directions, respectively.

## 3. Results and discussion

### 3.1. Self-assembly behaviour of casein

**Fig 3:**
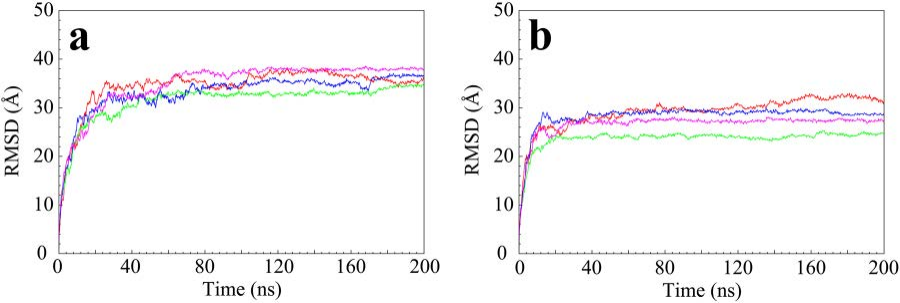
RMSD of the backbone of the four κ-casein molecules (a) and the four β-casein molecules (b).

**Fig 4:**
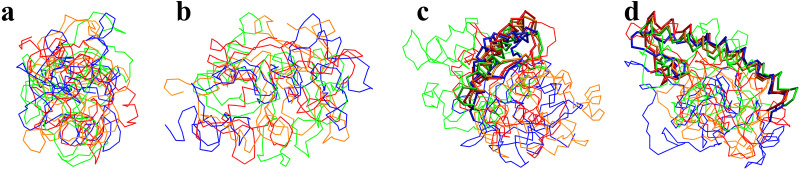
The structure of the CG κ-casein (a, b) and β-casein (c, d) in water. Different colors represent casein molecules. The κ-casein molecules are aligned as backbone particles, and the β-casein molecules are aligned as the backbone particles of the No. 16- 64th residues. (a) and (b) are the front and side views of κ-casein molecules, respectively, and (c) and (d) are the front and side views of β-casein molecules, respectively.

The stretched structures of four κ-casein and four β-casein molecules collapsed into a spherical shape after a 200 ns CG simulation. The root-mean-square displacements (RMSDs) of backbone particles (see Fig 3) show that all κ-casein and β-casein molecules reach stable structures after 200 ns simulations. Considering that κ-casein does not have a stable secondary structure, the four κ-casein molecules are aligned by the backbone particles. Figs 4a and 4b show the aligned structures for the four κ-casein molecules from front and side views. The β-casein structure predicted by AlphaFold has a stable helix of 49 residues at the N-terminal with a high confidence score. Our simulation also observed the stable helical structure. Figs 4c and 4d show the aligned structures for the four β-casein molecules from front and side views, which are aligned by the backbone particles of the helix. The aligned structures show that the spherical structures of the four spherical κ-casein molecules are different, but they are roughly the same size, and so are the four β-casein molecules.

### 3.2 Structural properties of the simulated κ-casein/β-casein aggregate

**Fig 5:**
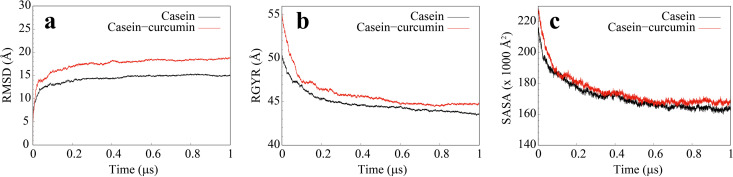
(a) RMSD of the backbone of the aggregate; (b) Radius of gyration of the aggregate; (c) Solvent accessible surface area of the aggregate. The black lines are for the casein system, and the red lines are for the casein-curcumin system.

**Fig 6:**
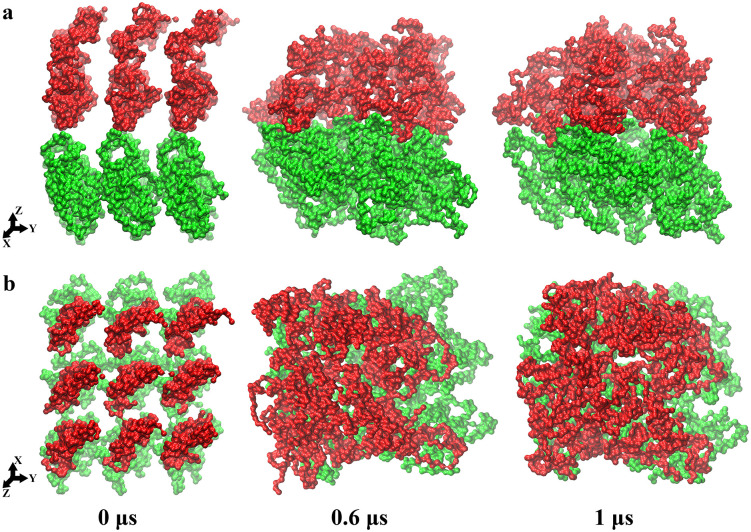
The snapshots of the aggregate self-assembly simulations. (a) and (b) are the side and top views of the aggregate. The κ-casein molecules are shown in red and β-casein in green.

κ-casein stabilizes micelle formation, preventing casein precipitation in milk, but the structure of the micelle is still unclear. In a 1 μs CG simulation, we have observed the heterogeneous structure of an aggregate composed of nine κ-casein and nine β-casein molecules (casein system). Fig 5a shows the RMSD of the aggregate, and a stable structure is formed after a 1 μs simulation. The radius of gyration (RGYR) decreases sharply in ~0.4 μs, then decreases slowly until it reaches ~44 Å (see Fig 5b) at the end of the simulation. The solvent accessible surface area (SASA) has undergone similar changes as the RGYR (see Fig 5c). The final value of SASA is ~165,000 Å^2^, which is significantly greater than the surface area of a sphere with a radius of 44 Å. Fig 6 shows the loose structure of the aggregate from the side and top views. The κ-casein and β-casein molecules attract each other to form an aggregate with a relatively loose structure, where κ-casein molecules on the top are smaller and therefore arrange more tightly than the β-casein molecules below. We also observed many water molecules in the cavity of the aggregate.

### 3.3 Encapsulation of the curcumin

**Fig 7:**
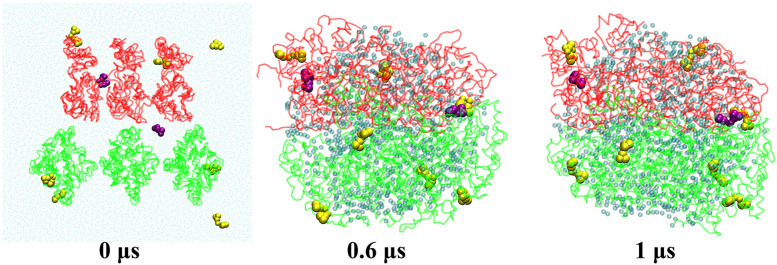
The snapshots of the casein-curcumin mixed aggregate self-assembly simulations. The transparent cyan beads are water inside the mixed aggregate; The κ-casein molecules are shown in red line and β-casein in green; The vdw beads are curcumin molecules, of which two purple ones are initially placed in the middle of casein molecules, and the remaining seven (yellow) have their initial positions in water.

As shown in Fig 7, nine curcumin molecules were randomly placed into the casein-curcumin system, with two (purple) located in the middle of the casein molecules and the remaining seven (yellow) are “outside” of the casein. After 1 μs self-assembly simulation, all curcumin molecules eventually adhered to the surface of casein molecules to form a mixed aggregate. Even the two middle curcumin molecules were not encapsulated inside the aggregates. We did not observe any spontaneous entry of curcumin into the interior of the aggregate. As same of casein aggregate, the structure of the mixed aggregate was also loose and contained many water molecules inside. The size and surface area of mixed aggregate were slightly larger than casein aggregate (see Fig 5) due to the attachment of curcumin molecules to the shallow surface.

### 3.4 The interaction between curcumin and casein aggregate

**Fig 8:**
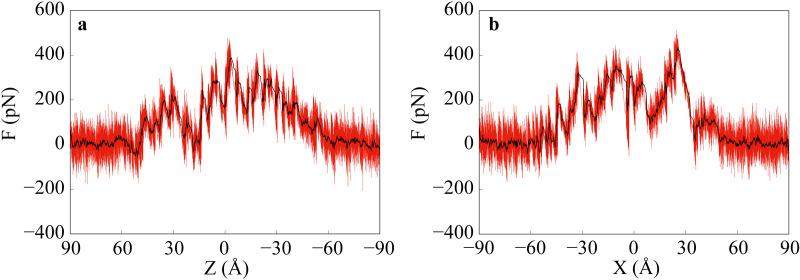
Force applied to the curcumin. The black line is the average of every 50 data points. (a) and (b) are perpendicular- (Z-direction) and parallel-direction (X-direction) systems respectively.

**Fig 9:**
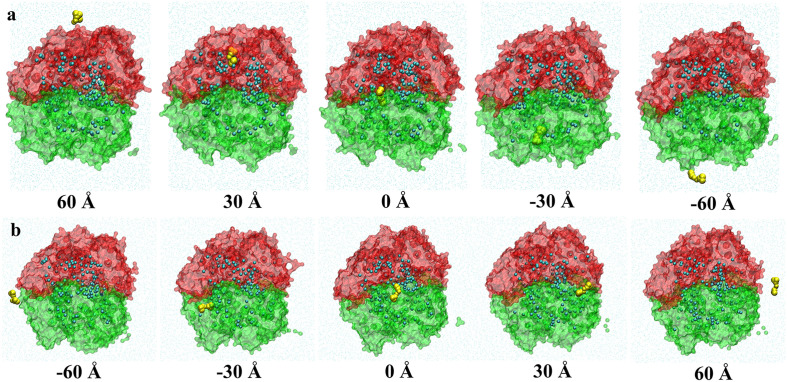
The snapshots of curcumin passing through the casein aggregate along the direction to which are perpendicular (a) and parallel (b), respectively. The center of the aggregate was at the origin, the curcumin molecule was pulled along the Z-direction (a) and X-direction (b), respectively. The cross-sectional views are displayed for clarity. The surface of the aggregates was drawn in transparent red for κ-casein and green for β-casein; Some water beads inside the aggregates were drawn in cyan, and the curcumin was drawn as yellow beads.

We carried out two SMD simulations to study the interaction between curcumin and casein aggregate. A curcumin molecule was pulled along the direction perpendicular and parallel to the bilayer to pass through the casein aggregate at a constant velocity of 0.005 Å/ps, respectively. In the perpendicular-direction system, the initial position of the curcumin molecule was ~40 Å above the top layer of κ-casein (Z-direction), which ensured that curcumin was far enough from the aggregate to avoid interaction. In the parallel-direction system, the initial position was placed ~40 Å away from the X-direction of the bilayer. Fig 8 shows the variation of the force applied to the curcumin molecule. The curcumin molecule was pulled from 90 to -90 Å along the Z-direction (Fig 8a), and it passed through the κ-casein and β-casein layer in sequence. The force was weak before the curcumin reached the position of ~50 Å, where it began to interact with the κ-casein molecules. Then, the strength of a downward force increased sharply because of the hindrance of tightly arranged κ-casein molecules. The local maximum value of force occurred at ~30 Å, where the curcumin was entering a cavity of the aggregate. We observed many cavities with water molecules inside. Fig 9 shows snapshots of curcumin at five typical positions. The strength of the force gradually weakened in the cavity, and then increased again as the curcumin was about to leave the cavity at ~20 Å until it reached the interface between κ-casein and β-casein. Afterwards, the strength of the force gradually weakened until the curcumin left the aggregate and completely entered bulk water. In the parallel direction (Fig 8b), the curcumin passed through the interface between κ-casein and β-casein. The strength of the force was greater in the aggregate, while it weakened when the curcumin entered a certain cavity. The results of SMD simulation indicate that it is difficult for the curcumin, which is insoluble in water, to enter the aggregate from the κ-casein side, and it cannot spontaneously leave the aggregate from the β-casein side or the interface between κ-casein and β-casein, even if there are many cavities inside the aggregate.

In addition, we observed many water particles entering or exiting the cavity during the passage of curcumin through the aggregate. Due to computational limitations, we only simulated an aggregate composed of eighteen casein molecules, while the real micelles are composed of a large number of caseins. It can be inferred that curcumin is difficult to spontaneously enter or exit the casein micelle.

## 4. Conclusions

In this study, the formation of κ-casein/β-casein aggregates and their interaction with curcumin were investigated using computational models and simulations. Compared to the all-atom model, the MARTINI CG model provides a more efficient method to simulate large systems. In a recent all-atom Hamiltonian replica exchange molecular dynamics simulation study, the calculated free-energy landscape of β-casein conformations contains a global minimum [37]. Around the global minimum, the conformations are globular and collapsed [37], which is consistent with our results. The all-atom simulations also indicate that the internal compactness of β-casein exhibits strong conformational heterogeneity [37]. In current CG simulations, the self-assembly of κ-casein and β-casein molecules into an aggregate was studied, reaching a stable structure after 1 μs of simulation. The aggregate contained many cavities filled with water, which may be attributed to the conformational heterogeneity of β-casein. In addition, we probed the self-assembly behavior of a mixed molecule system containing casein and curcumin. All curcumin molecules adhered to the surface of the casein aggregate and cannot be spontaneously encapsulated by casein.

The interaction between the aggregate and curcumin was also explored using SMD, showing that it is difficult for curcumin to enter the aggregate without external drag due to the tight arrangement of κ-casein molecules. The results suggest that water and insoluble substances such as curcumin may find it challenging to enter or exit casein micelles. Therefore, external forces are required to facilitate the interaction between curcumin and caseins to achieve effective encapsulation. This understanding is practically important for utilizing caseins as carriers of bioactive compounds. However, the precise binding location of curcumin within casein micelles remains unclear and warrants further investigation. More comprehensive studies are needed to elucidate the encapsulation mechanism, including the specific interaction forces involved, the distribution of curcumin between the surface and interior of casein micelles, and changes in the micelles’ surface properties. These factors critically influence the functional properties of casein micelles, such as rennet coagulation, acid-induced gelation, and water-binding capacity.

## Abbreviations

CGMD: Coarse-grained molecular dynamics
RMSD: Root-mean-square displacements
VMD: Visual Molecular Dynamics
SMD: Steered Molecular Dynamics
